# Comparison of 10 biometric formulas in combined phacovitrectomy for different underlying retinal pathology

**DOI:** 10.1038/s41598-025-97839-x

**Published:** 2025-04-17

**Authors:** Eleftherios Chatzimichail, David L. Cooke, Christian Wertheimer, Susanna Koenig, Zisis Gatzioufas, Armin Wolf, Efstathios Vounotrypidis

**Affiliations:** 1 Department of Ophthalmology, University Hospital Ulm, Prittwitzstr. 43, 89075 Ulm, Germany; 2 Department of Ophthalmology, University Hospital Basel, Basel, Switzerland; 3 Great Lakes Eye Care, St. Joseph, MI USA; 4 Department of Neurology and Ophthalmology, College of Osteopathic Medicine, Michigan State University, East Lansing, MI USA

**Keywords:** Phacovitrectomy, Refractive outcome, Biometric formula, Retinal pathology, Cataract, Outcomes research, Medical research, Phase IV trials

## Abstract

The refractive accuracy of intraocular lens (IOL) formulas varies in eyes that undergo combined phacovitrectomy for different underlying vitreoretinal pathology. A total of 401 eyes that underwent uncomplicated phacovitrectomy (23–25 g) with implantation of a plate haptic IOL (CT Asphina 409 M) in the capsular bag between April 2020 and December 2022 were included in the study. Inclusion criteria were postoperative best corrected visual acuity of 0.4 LogMAR or better at least 8 weeks after surgery. The Barrett Universal II (BUII), Haigis, Hill-Radial Basis Function (Hill-RBFv3.0), Hoffer Q, Holladay I, Holladay II, Kane, K6, Pearl-DGS and SRK/T formulas were compared for their accuracy in mean absolute error (MAE). Furthermore, all formulas were additionally tested by using the prediction for an IOL power 0,5 or 1D above the IOL used (IOLdown). Wilcox-Holladay-Wang-Koch (WHWK) statistical tests with Holm correction were applied. The Barrett IOLdown formula showed the lowest refractive prediction error (0.03D; together with Hill IOLdown), lowest MAE (0.40D), lowest median absolute error (MedAE; 0.31D), lowest standard deviation of MAE (0.55D) and lowest root mean squared absolute error (RMSAE; 0,54D). Barrett IOLdown had the highest percentage of eyes with predicted error within ± 0.25D (41.6%), Kane IOLdown within ± 0.50D (70.5%) and ± 0.75D (89%) and Hill IOLdown within ± 1D (95.2%). Except for Haigis, Hoffer Q and Pearl-DGS, all other formulas showed a statistically significantly lower MAE after IOLdown modification. The newest formulas with IOLdown modification performed better than old generation formulas, with Barrett IOLdown exhibiting the best results.

**Supplementary Information:**

The online version contains supplementary material available at 10.1038/s41598-025-97839-x.

## Introduction

Phacovitrectomy has been increasingly popular over the last decade, combining phacoemulsification with pars-plana vitrectomy and IOL-implantation. The advantages of the combined procedure have been reported in a plethora of studies, including better intraoperative visualization, faster recovery and avoiding a subsequent operation allowing for overall lower therapy costs^1^.

The technological advance has increased the expectations of both surgeons and patients for precise results after surgery. Reported data differ strongly, with several studies having shown a myopic shift, while other studies reported no myopic shift after phacovitrectomy^2^. Therefore, calculating the optimal lens power for phacovitrectomy still remains challenging. Recently, a new methodology with an IOL-Power adjustment was proposed to avoid hyperopic shift in eyes undergoing triple DMEK^3,4^. To the best of our knowledge, however, no corresponding methodology has yet been investigated in eyes that underwent combined phacovitrectomy.

Over the last few years, a variety of new formulas to calculate lens power have been developed^5^. There are many published studies on comparison of formulas for phacoemulsification; recently more and more studies attempt to compare the performance of different biometric formulas as well as the refractive outcomes after combined phacovitrectomy^1^. However, except for one prospective study all others include either a small number of eyes, different IOL models or different biometers.

Our study is the largest one that compares 10 different biometric formulas (Barrett Universal II, Haigis, Hill-RBF, Holladay 1, Holladay 2, Hoffer Q, Kane, K6, Pearl-DGS and SRK-T) in combined phacovitrectomy for four different underlying retinal pathologies (rhegmatogenous retinal detachment, epiretinal membrane, macular hole, vitreomacular traction syndrome) with a swept-source biometer and implantation of one IOL model. Furthermore, this is the first study that investigates the IOL-Power modification method in terms of combined phacovitrectomy and as suggested by newest studies, we compared separately 6 formulas in eyes with previous refractive surgery^6,7^.

## Results

A total of 401 consecutive eyes (208 right) of 401 patients (162 female) that underwent uneventful combined small gauge phacovitrectomy with implantation of the same plate haptic IOL due to different underlying pathology between 01.04.2020 and 31.12.2022 were included in the study. Demographics of both groups are shown in Table 1. Underlying retinal pathology was epiretinal membrane (ERM) in 132 eyes, macular hole (MH) in 90 eyes, rhegmatogenous retinal detachment (RRD) in 144 eyes (72 with detached macula) and 35 eyes suffered from vitreomacular traction syndrome (VMTS). Forty-five eyes had undergone refractive surgery previously (11.2%).

**Table 1:** Demographics and preoperative biometric data. SD: standard deviation, K1: flat meridian, K2: steep meridian, ACD: anterior chamber depth, LT: lens thickness, W2W: horizontal corneal diameter (white-to-white), IOL: intraocular lens, Mm: millimetre, D: Dioptre.

Parameter	Mean ± SD	Range
Age (years)	67 ± 8.5	40–88.3
Axial length (mm)	24.36 ± 1.65	20.60–32.59
K1 (D)	42.31 ± 1.60	37.47–47.15
K2 (D)	43.10 ± 1.62	38.83–47.99
ACD (mm)	3.23 ± 0.43	2.00–4.39
LT (mm)	4.64 ± 0.43	3.59–6.64
W2W (mm)	12.13 ± 0.40	10.70–13.70
IOL-Power (D)	19.09 ± 3.83	2–32

**Fig. 1:**
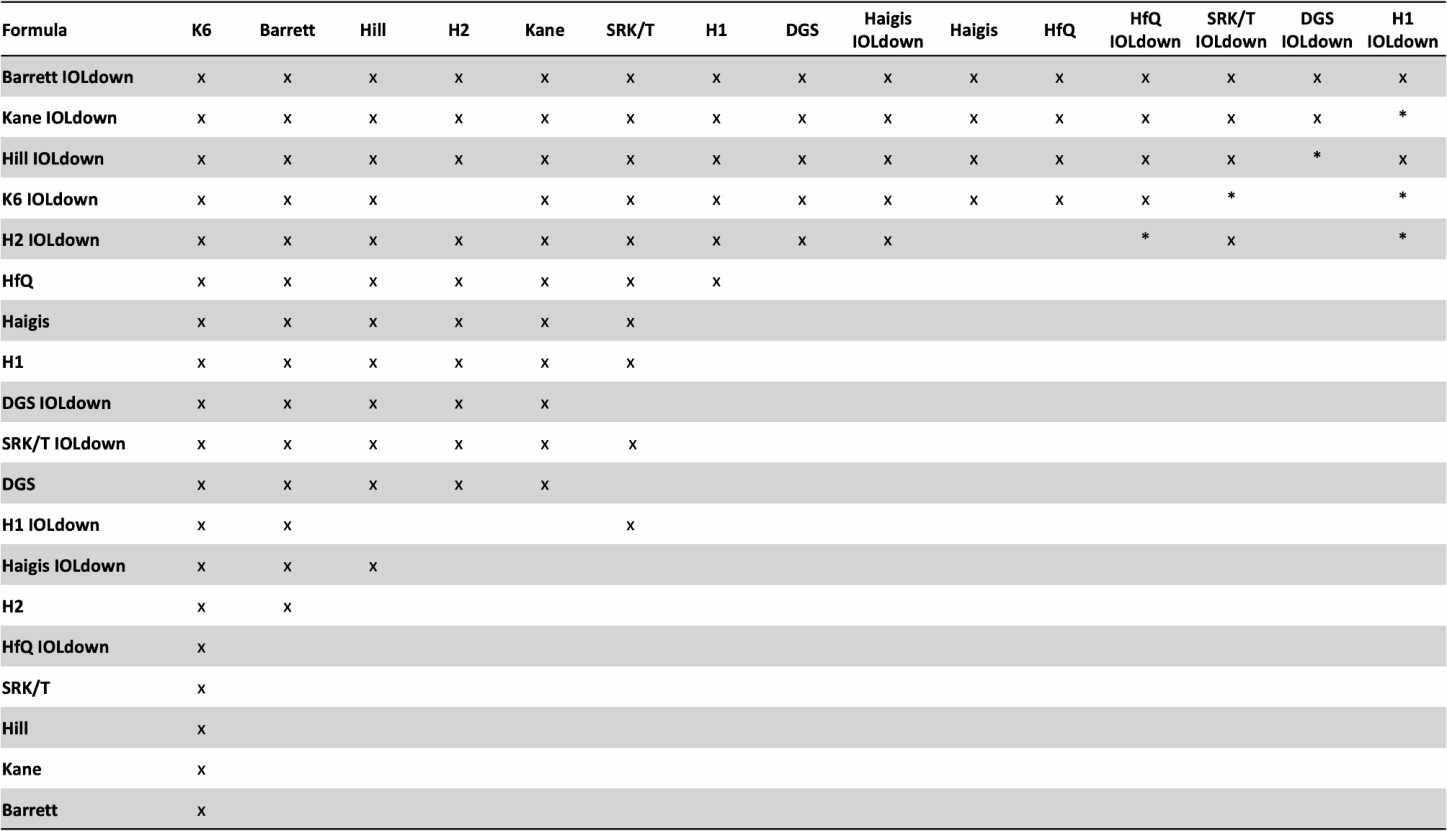
Summary table of significance for comparison between formulas with implanted IOL power and “IOL down” modification using WKWH MAE test with Holm’s correction. The top formula was significantly worse than the formula in the column to the left by the p-value listed. P-value was zero when marked with “x” and between 0.001 and 0.05 when marked with *. IOL: intraocular lens; WKWH: Wilcox-Holladay-Wang-Koch; MAE: mean absolute error; H1: Holladay 1; H2: Holladay 2; HfQ: Hoffer Q; K6: Cooke K6; DGS: Pearl-DGS; Hill: Hill RBF v.3.0.

All formulas with the exception of Hoffer Q showed an improvement after the IOL down modification. This was statistically significant for all formulas except for Haigis and DGS, with DGS exhibiting a near significant outcome (*p* = 0.056; Table 2). Barrett IOL down showed the lowest mean refractive prediction error, lowest mean absolute error, lowest median absolute error, lowest SD of MAE and lowest RMSAE. Kane IOL down, Hill IOL down, K6 IOL down and Holladay IOL down showed comparable results, and no statistically significant difference was found between these formulas with regard to MAE. Interestingly Kane IOL down showed the highest percentage of eyes within 0,5D and 0,75D, whereas Hill showed the highest percentage within 1D. However, these differences are comparable and clinically insignificant among the aforementioned formulas. Overall, Barrett, followed by Kane, Hill, K6 and Holladay 2, all with IOL down modification showed the best performance (Table 3). Figure 1 shows the statistically significant comparisons between employed formulas (original and IOLdown).

**Table 2:** Table of comparisons between original formulas and IOL down modification with p-values, showing that all formulas, except for hoffer Q, were better with IOL down modification. Formula 1 is better than formula 2 by the p-value provided in the right column. H1: Holladay1, H2: Holladay2, HfQ: hoffer Q, DGS: Pearl-DGS, IOLdown: IOL down modification.

Formula 1	Formula 2	*p*-value
Barrett IOLdown	Barrett Original IOL	0000
Hill-RBF 3.0 IOLdown	Hill-RBF 3.0 Original IOL	0.000
Holladay 2 IOLdown	Holladay 2 Original IOL	0.000
Kane IOLdown	Kane Original IOL	0.000
K6 IOLdown	K6 Original IOL	0.000
SRK/T IOLdown	SRK/T Original IOL	0.000
H1 IOLdown	H1 Original IOL	0.042
DGS IOLdown	DGS Original IOL	0.056
Haigis IOLdown	Haigis Original IOL	0.793
HfQ Original IOL	HfQ IOLdown	0.878

**Table 3:** All formulas ranked by MAE. Mean refractive prediction error (MRE), mean absolute error (MAE) and median absolute error (MedAE), standard deviation of absolute error (SD), root mean squared of absolute error (RMSAE) as well as percentage of eyes within 0.25D, 0.5D, 0,75D and 1D with regard to the biometric formula. H1: Holladay1, H2: Holladay2, HfQ: hoffer Q, DGS: Pearl-DGS, IOLd: IOL down modification. Best values are marked bold.

Formula	MRE	MAE	Med AE	SD	% +/-0.25 D	% +/- 0.5 D	% +/- 0.75 D	% +/- 1.0 D	RMS AE
Barrett IOLd	**0**.**03**	**0**.**40**	**0**.**31**	**0**.**55**	**41**.**6%**	69.7%	87.4%	94.7%	**0**.**54**
Kane IOLd	0.04	0.41	0.32	0.55	39.9%	**70**.**5%**	**89**.**0%**	94.4%	0.55
Hill RBF 3.0 IOLd	0.03	0.41	0.32	0.55	39.3%	69.1%	87.6%	**95**.**2%**	0.55
K6 IOLd	-0.05	0.41	0.33	0.56	37.9%	70.2%	85.4%	94.9%	0.56
H2 IOLd	0.07	0.42	0.36	0.56	38.2%	66.0%	86.2%	94.4%	0.57
DGS IOLd	0.22	0.45	0.38	0.56	36.5%	63.2%	82.9%	92.4%	0.61
H1 IOLd	0.19	0.46	0.38	0.59	35.7%	61.5%	84.6%	93.0%	0.62
SRK/T IOLd	0.13	0.46	0.37	0.60	33.7%	62.9%	84.3%	90.4%	0.62
Haigis IOLd	0.24	0.47	0.40	0.57	32.0%	61.0%	82.6%	94.1%	0.61
HfQ	-0.22	0.47	0.38	0.58	35.1%	63.5%	80.9%	89.6%	0.62
Haigis	-0.25	0.47	0.38	0.56	32.9%	62.4%	81.2%	88.2%	0.62
HfQ IOLd	0.25	0.47	0.40	0.58	33.7%	60.4%	81.5%	92.4%	0.63
DGS	-0.27	0.49	0.40	0.57	30.3%	61.0%	79.2%	91.0%	0.63
H1	-0.28	0.50	0.40	0.60	34.0%	61.0%	77.2%	87.9%	0.66
SRK/T	-0.34	0.55	0.44	0.61	27.8%	56.5%	73.6%	85.1%	0.70
H2	-0.39	0.55	0.46	0.57	29.5%	53.7%	74.4%	85.4%	0.69
Hill RBF 3.0	-0.44	0.56	0.50	0.55	32.3%	50.0%	71.9%	84.8%	0.70
Kane	-0.43	0.56	0.50	0.56	28.1%	50.3%	71.1%	85.4%	0.70
Barrett	-0.46	0.58	0.52	0.55	27.8%	48.3%	68.3%	84.6%	0.72
K6	-0.51	0.61	0.56	0.56	26.1%	45.8%	67.1%	80.9%	0.76

**Fig. 2:**
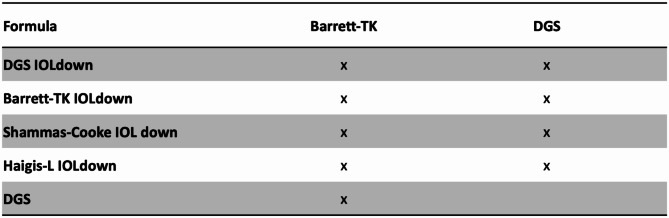
Summary table of significance for comparison between formulas with implanted IOL power and “IOL down” modification using WKWH MAE test with Holm’s correction. The top formula was significantly worse than the formula in the column to the left by the p-value listed. P-value was zero when marked with “x”. There were no statistically significant relationships between following formulas: DGS IOLd – Shammas-Cooke IOLd, DGS IOLd – Barrett-TK IOLd, DGS IOLd – Haigis-L IOLd, Barrett-TK IOLd – Shammas-Cooke IOLd, Barrett-TK IOLd – Haigis-L IOLd, Shammas-Cooke IOLd – Haigis-L IOLd. IOL: intraocular lens; WKWH: Wilcox-Holladay-Wang-Koch; MAE: mean absolute error; DGS: Pearl-DGS; Barrett-TK: Barrett-True K; IOLd: IOL down modification.

The subanalysis regarding previous refractive surgery showed different results. The detailed results are presented in Table 4. All refractive surgery calculations had an additional diopter of IOL down power than would have otherwise been applied for retinal pathology. Overall, in eyes with previous refractive surgery DGS IOL down showed the lowest MAE, MedAE, RMSAE and highest percentages within 0,5D, 0,75D and 1D. Barrett True-K, Shammas-Cooke and Haigis-L, all with IOL down modification, showed slightly worse results, though not statistically significant. All four formulas showed statistically significant better results than Barrett True-K and DGS with original IOL selection. Figure 2 shows the statistically significant comparisons between employed formulas.

**Table 4:** Table of M-LVC eyes, ranked by MAE. Note the large difference in mean error between using the original DGS and Barrett formulas and using formula versions specifically designed for post-MLVC (the first four rows) combined with IOL down method. Mean refractive prediction error (MRE), mean absolute error (MAE), median absolute error (MedAE), standard deviation of absolute error (SD), root mean squared of absolute error (RMSAE) as well as percentage of eyes within 0.25D, 0.5D, 0,75D and 1D with regard to the biometric formula. M-LVC: myopic laser vision correction, DGS: Pearl-DGS, IOLd: IOL down adjustment, Barrett-TK: Barrett-True K. Best values are marked bold.

Formula	MRE	MAE	Med AE	SD	% +/- 0.25 D	% +/- 0.5 D	% +/- 0.75 D	% +/- 1.0 D	RMSAE
DGS IOLd	0.07	**0**.**49**	0.33	0.66	28.9%	**68**.**9%**	**80**.**0%**	**88**.**9%**	**0**.**655**
Barrett-TK IOLd	-0.29	0.53	0.44	**0**.**65**	**37**.**8%**	57.8%	77.8%	84.4%	0.70
Shammas-Cooke IOLd	**0**.**03**	0.59	0.48	0.74	26.7%	53.3%	66.7%	80.0%	0.73
Haigis-L IOLd	-0.16	0.55	0.47	0.72	33.3%	55.6%	73.3%	82.2%	0.73
DGS	-1.33	1.35	1.28	0.67	4.4%	11.1%	13.3%	22.2%	1.48
Barrett-TK	-1.62	1.62	1.57	0.64	2.2%	4.4%	11.1%	13.3%	1.74

Additional results with regard to the specific underlying pathology (ERM, MH, RRD, VMTS) are provided as supplemental material.

## Discussion

The refractive outcomes of combined phacovitrectomy have demonstrated notable improvement in recent years, attributed to advancements in surgical techniques, enhanced diagnostic precision^8^ and the introduction of innovative formulas^1,9^. However, most published studies have included a limited number of patients or employed different IOL models. While new knowledge regarding the effect of the applied endotamponade is taken into account^10–12^, larger studies are necessary to determine how to minimise the refractive prediction error, achieve favourable outcomes and provide adequate information to the patient preoperatively. Therefore, this study was conducted to investigate the refractive accuracy of ten biometric intraocular lens formulas in eyes that have undergone combined phacovitrectomy for different vitreoretinal conditions. This study enrolled eyes with four different pathologies (VMT, ERM, MH, RRD), used a single IOL model, included the largest number of patients to date, and compared ten different biometric formulas, representing both old and new generation. Furthermore, as recently proposed for other combined surgical procedures, we employed the IOL down method and evaluated its effect in the refraction error in terms of phacovitrectomy.

Previous studies from our research group demonstrate a statistically significantly superior performance of the Kane and Barrett formulas in phacoemulsification, along with Kane formula in the context of combined phacovitrectomy with ILM peeling^1^. Tanaka et al. conducted a similar case study involving a smaller sample size of 104 eyes, divided into three groups: those undergoing phacoemulsification alone, phacovitrectomy for epiretinal membrane (ERM), and phacovitrectomy for rhegmatogenous retinal detachment (RRD)^9^. This study evaluated the accuracy of three biometric formulas: SRK-T, Hill-RBF, Kane, and Barrett across these different surgical approaches. Lowest mean prediction error between phacovitrectomy groups was in the phacovitrectomy for epiretinal membrane. Barrett formula exerted the lowest MPE and MAE without significant differences.

A similar study from 2021, involving 202 eyes, reported superior performance of the Kane formula, demonstrating lower SD, MAE, and median absolute error (MedAE) in the phacovitrectomy group. Across all three groups of the study, the Kane formula consistently demonstrated the highest accuracy, followed by Pearl-DGS^13^. In a large retrospective study with 357 eyes, Choi et al. compared the outcomes of four old generation biometric formulas with one IOL: Hoffer Q, Holladay 1, Holladay 2, and SRK-T^2^. Hoffer Q and Holladay 1 showed the best performance in terms of both mean absolute refractive error and mean refractive erect, but without any significant differences among the four biometric formulas.

More recently, Thanitcul et al. evaluated the refractive outcomes from 59 eyes that underwent uneventful phacovitrectomy^14^. The authors compared several biometric formulas, including Barrett Universal II (BUII), Emmetropia Verifying Optical (EVO v2.0), Hill-Radial Basis Function (Hill-RBF v3.0), Hoffer Q, Holladay 1, Kane, Ladas Super Formula (LSF), and SRK/T. Their analysis revealed the superiority of the BUII formula, which produced the lowest mean error (-0.043D), mean absolute error (MAE) of 0.39D, and median absolute error (MedAE) of 0.23D. Furthermore, the BUII formula had the highest percentage of eyes with a predicted error within ± 0.25 D (51%) and ± 0.50 D (83%).

This study showed statistically significant improvement of all employed formulas except of Haigis, Hoffer Q and DGS after the application of IOL down modification, with DGS nearly reaching significance. Based on previous findings, as well as on our results, we adjusted the IOL power 0.5D for eyes with ERM, VMTS or MH and 1D for eyes with RRD as these eyes tend to show a higher refractive shift^1,8^.

In eyes with previous refractive surgery, prior studies have examined these formulas only in cataract eyes. For example, Rong et al. analysed 78 eyes that had undergone previous myopic refractive surgery, comparing 12 biometric formulas, and found that the EVO and Barrett True-K formulas demonstrated superior performance in terms of mean absolute error^15^. Further studies also support the use of Barrett True-K formulas and Barrett True-K No History in eyes with prior refractive surgery^16–18^. In order to follow recent published data^7^, we employed biometric formulas with higher accuracy for eyes after refractive surgery such as Shammas-Cooke, Haigis L, Barrett True-K and Pearl-DGS formulas. While all four formulas with IOL down modification showed comparable results, and statistically significantly better than DGS or Barrett True-K without IOL modification, we still observed higher refractive PE compared to the rest of the eyes.

This may be attributable to the fact that most of the eyes with previous refractive surgery underwent phacovitrectomy due to RRD, a category with the least precision. Nevertheless, the majority of the formulas performed better after IOL down modification, indicating the importance of this implementation in the everyday practice.

Although the underlying retinal disease appears to influence the final refractive outcome (supplemental material), it is established that accurate preoperative parameters are essential for precise IOL power calculation^19,20^. Olsen reported that measurement inaccuracies in axial length and anterior chamber depth (of the IOL) can contribute up to 36% and 42% of errors in IOL power calculations, respectively^21^. In contrast, Norrby reported lower error contributions of 17% and 25% for these parametes in an eye with average dimensions^22^.

In the present study, patients with ERM exhibited a slight myopic shift, which was more pronounced in the MH group and even more so in the RRD group. In contrast, patients with VMTS exhibited a much lower tendency towards myopia. Previous studies have demonstrated a myopic shift following phacovitrectomy for ERM^1,8^. Conversely, other studies have not identified any significant difference between ERM and VMTS^23^. Despite the absence of an impact on postoperative ACD resulting from the type of endotamponade employed in phacovitrectomy^10–12^, all cases that received gaseous endotamponades (MH and RRD) demonstrated a higher myopic shift in comparison to cases that received air or BSS endotamponade (ERM or VMTS). The precise reason for this finding remains unclear. However, it may be attributed to a combination of factors, including surgeon preference, the use of gaseous endotamponade, and potential issues with patient fixation in these cases.

In terms of the limitations of this study, its retrospective nature represents the most significant issue. Furthermore, the utilisation of different endotamponades during vitrectomy represents another potential drawback, which may have resulted in multifactorial differences in the observed outcomes. As only two surgeons (EV, AW) performed all surgeries, this constitutes an additional possible constraint factor. Conversely, several factors strengthen the rigour of this study. The present study comprises a substantial number of patients and compares ten different biometric formulas (Barrett, Haigis, Hill-RBF, Holladay 1, Holladay 2, Hoffer Q, Kane, K6, Pearl-DGS, and SRK-T). To the best of our knowledge, this is the first study to compare such a wide array of biometric formulas with a large sample size in terms of phacovitrectomy. Additionally, the inclusion of a wide range of retinal pathologies (rhegmatogenous retinal detachment, epiretinal membrane, macular hole, and vitreomacular traction syndrome) alongside the use of a single intraocular lens (IOL) model serves to further enhance its robustness. Moreover, given that most surgeons do not adjust their constants for phacovitrectomy, we propose the simple IOL down method in order to achieve even truer results in these cases.

In conclusion, our data indicate that all newest biometric formulas with IOL down modification (0,5D for ERM, MH or VMTS and 1D for RRD case) yielded favourable outcomes in phacovitrectomy for diverse underlying retinal pathologies. Post LVC formulas with IOL down modification by 1D showed better results than with original IOL selection independent from the underlying retinal disease. Barrett formula with IOL down modification exhibited a slight superiority across the entire study population. We suggest that surgeons adapt their results according to the specific formulas they employ and consistently utilise the same IOL models to enhance precision. Furthermore, future AI models may offer the potential to further enhance outcomes by refining predictions based on specific diagnoses. Further studies with larger sample sizes, more biometric formulas and a broader range of preoperative biometric parameters are required to validate and generalise the findings of this study.

## Methods

### Study design

This retrospective single-center study was carried out in the Department of Ophthalmology, Ulm University, Ulm, Germany and included eyes that underwent combined small-incision cataract surgery and small-gauge vitrectomy for age-related cataract and epiretinal membrane (ERM), macular hole (MH), rhegmatogenous retinal detachment (RRD) or vitreomacular traction syndrome (VMTS) between April 2020 and December 2022. We acquired a proper informed consent for the surgical procedure preoperatively from each subject. Further inform consent was waived due to the retrospective nature of the study. The study was approved by the local ethics committee of Ulm University (Ethic number: 36/22) and adhered to the tenets of the Declaration of Helsinki.

Prerequisites for enrolment were uneventful surgery with in-the-bag implantation of the same IOL, absence of intra- or postoperative complications, preoperative swept-source OCT-based biometry (IOL Master 700), follow-up period of eight weeks twelve weeks after surgery, postoperative CDVA ≤ 0.4 Logmar and corneal astigmatism ≤ 3D. Only one eye from each patient was included in the study. IOLCon-optimized constants were used for all calculations^24^. Refractive prediction error (PE), and absolute error and root mean squared of absolute errors were calculated for biometric formulas Barrett Universal II (BUII), Haigis, Hill-RBF 3.0., Holladay 1, Holladay 2, Hoffer Q, Kane, K6, Pearl-DGS and SRK-T. For eyes with previous refractive surgery Barrett True-K, Shammas-Cooke, Haigis-L and Pearl-DGS formulas were evaluated.

### Examinations

All subjects were examined preoperatively as well as postoperatively, 1 day and 8–12 weeks after surgery. Ophthalmological examination included past ocular history, objective refraction (Visuref-1000, Carl Zeiss Meditec AG), CDVA, Goldmann applanation tonometry, slit-lamp examination, optical biometry (IOLMaster700, Carl Zeiss Meditec AG), dilated fundoscopy and, when possible, macular OCT (Spectralis OCT, Heidelberg Engineering).

### Surgical technique

In all groups, subjects received a standard micro-incisional phacoemulsification with in-the-bag implantation of a hydrophilic 1-piece plate-loop posterior chamber IOL (CT Asphina 409 M, Carl Zeiss Meditec AG) and a 23–25 g pars plana vitrectomy (OS4, Oertli) including core vitrectomy, posterior vitrectomy, and vitreous base shaving. In cases of ERM, MH and when necessary, in RRD, membrane and/or ILM-peeling using brilliant blue dye and Eckart forceps was performed. Retinal endolaser was applied in all cases of RRD and when necessary, in the rest of the cases. Balanced salt solution (BSS), air and gas (either SF6 20% or C2F6 15%) served as endotamponade depending on underlying pathology and other individual factors, such as surgeons’ preference. Two surgeons performed all surgeries (AW, EV). Postoperative treatment included topical antibiotics for 5 days and topical steroids 6–8 times daily, tapered by 1 eye drop per week.

### Biometric data assessment, refractive error calculation and OCT verification

Swept-source OCT-based biometry with the IOL Master 700 (Carl Zeiss meditec, Germany) was preoperatively performed in all cases and according to the manufacturer’s guidelines, including an internal integrity check. Cases of RRD with macular involvement were included only if internal integrity check was given and controlled by means of fellow eye and in some cases confirmed with ultrasound. Only eyes with an AL-difference ≤ 0,2 mm (Axial length difference between Swept-Source OCT-based biometry and ultrasound) were included in the study. Eyes, where axial length could not be measured with the IOLMaster700 due to highly bullous RRD were excluded from the study.

The standard constants from the IOLCon website^24^ were utilised for the employed formulas. Moreover, as previous data have demonstrated a clear myopic shift after phacovitrectomy with this IOL^1^, we employed a method that has been recently proposed in order to further reduce the refractive PE^3,4^. This novel method involves the adjustment of IOL power selection by 1 dioptre to equalise the postoperative hyperopic shift of 0.75-1D in eyes undergoing triple DMEK. This approach is intended to target myopia to achieve emmetropia. In the context of IOL calculations performed prospectively, it is necessary to determine the target refraction and subsequently reduce the IOL power (less power). Conversely, for the purposes of this retrospective study, the reverse approach is taken. Given the known postoperative outcome and the already-implanted IOL, an IOL with + 0.5D or + 1.0D (higher power) is utilised. Due to the retrospective nature of this study, the “IOLdown” analysis calculates the target refraction of an IOL with 0.5D or 1.0D more power than the IOL implanted. In accordance with that, and based on our results, we adjusted for 0,5D down in eyes with ERM, MH and VMTS to equalise the observed myopic shift, and for 1D down in eyes with RRD. For refractive prediction error (PE), absolute error (AE) and root mean squared of absolute error (RMSAE), we calculated the postoperative spherical equivalent of objective refraction (ORSE). The refractive prediction error (PE) was calculated as the difference between postoperative refractive outcome expressed as the spherical equivalent and refraction predicted for each formula. A positive value of the PE indicates a hyperopic PE that corresponds to a more hyperopic result than the predicted refraction, whereas a negative PE shows a myopic shift.

The biometric formulas Haigis, Hoffer Q, Holladay1, SRK-T and Haigis-L were calculated in validated Excel Spreadsheets and calculations were performed with them. The Barrett Universal II and Hill-RBF (Version 3.0) were run within the EyeSuite software of the Lenstar biometer (Haag-Streit Diagnostics, EyeSuite i8.0.0.0, Haag-Streit AG). K6 and Pearl-DGS formulas were performed by their authors (David Cooke, and Guillaume Debellemanière – personal communication, February 2025). Shammas-Cooke and DGS for laser-vision-correction (LVC) were also performed by these same authors, respectively. Kane and Barrett True-K (no history) are available online (https://www.iolformula.com/ and https://calc.apacrs.org/Barrett_True_K_Universal_2105/, respectively) and the online portal was used for predictions. Holladay consultant (HicSoapPro, version 2021.1.1105) was used for predictions of the Holladay 2 formula.

The mean absolute error was calculated as the absolute value of the PE. The root mean squared of absolute error was calculated as previously described^25^. The percentages of the eyes with a refractive prediction error within ± 0.25 diopters (D), ± 0.5D, ± 0,75D and ± 1D were calculated.

### Statistical analysis

The MAE and RMSAE heteroscedastic tests given in the Wilcox-Holladay-Wang-Koch (WHWK) statistics package for R programming (osf.io/xhe8u/) were used for statistical comparisons between all formulas, including standard calculations and IOL down calculations for all formulas, as currently suggested^26^. Sample size calculation resulted in a total of 326eyes for an effect size of 0,25, 10 groups with one covariate factor (biometric formula), estimated power of 90% and an alpha error of 0.05.

## Electronic supplementary material

Below is the link to the electronic supplementary material.

Supplementary Material 1

## Data Availability

The dataset generated and analysed during the current study is available from the corresponding author on reasonable request.
